# Epigallocatechin-3-gallate Synergistically Enhanced Arecoline-Induced Cytotoxicity by Redirecting Cycle Arrest to Apoptosis

**DOI:** 10.3390/cimb46020098

**Published:** 2024-02-14

**Authors:** Li-Jane Shih, Po-Chi Hsu, Chih-Pin Chuu, Hao-Ai Shui, Chien-Chih Yeh, Yueh-Chung Chen, Yung-Hsi Kao

**Affiliations:** 1 Department of Medical Laboratory, Taoyuan Armed Forces General Hospital, Longtan, Taoyuan 325208, Taiwan; shihlijane@gmail.com (L.-J.S.); yeh3470@gmail.com (C.-C.Y.); 2 Department of Life Sciences, National Central University, Jhongli, Taoyuan 320317, Taiwan; 3 Institute of Cellular and System Medicine, National Health Research Institutes, Zhunan, Miaoli 350401, Taiwan; 4 Graduate Institute of Medical Science, National Defense Medical Center, Taipei 114201, Taiwan; 5 Division of Cardiology, Department of Internal Medicine, Taipei City Hospital, Renai Branch, Taipei 106243, Taiwan; chenyuehchung.tw@yahoo.com.tw

**Keywords:** epigallocatechin-3-gallate, arecoline, synergistic effect, cytotoxicity, cell cycle arrest, reactive oxygen species

## Abstract

Carcinogens, such as arecoline, play a crucial role in cancer progression and continuous gene mutations by generating reactive oxygen species (ROS). Antioxidants can reduce ROS levels and potentially prevent cancer progression but may paradoxically enhance the survival of cancer cells. This study investigated whether epigallocatechin-3-gallate (EGCG), an antioxidant from green tea, could resolve this paradox. Prostate cancer cells (PC-3 cell line) were cultured and treated with arecoline combined with NAC (N-acetylcysteine) or EGCG; the combined effects on intracellular ROS levels and cell viability were examined using the MTT and DCFDA assays, respectively. In addition, apoptosis, cell cycle, and protein expression were investigated using flow cytometry and western blot analysis. Our results showed that EGCG, similar to NAC (N-acetylcysteine), reduced the intracellular ROS levels, which were elevated by arecoline. Moreover, EGCG not only caused cell cycle arrest but also facilitated cell apoptosis in arecoline-treated cells in a synergistic manner. These were evidenced by elevated levels of cyclin B1 and p27, and increased fragmentation of procaspase-3, PARP, and DNA. Our findings highlight the potential use of EGCG for cancer prevention and therapy.

## 1. Introduction

The generation of reactive oxygen species (ROS) by various carcinogens, such as arecoline, can lead to damage in lipids, proteins, and DNA. ROS initiate cancer development through DNA base oxidation, gene mutations, oncogene activation, and tumor suppressor gene inhibition [1,2]. ROS-induced damage promotes the transition of benign cancer cells into malignant ones by excessively activating epithelial–mesenchymal transition signaling pathways [3]. Arecoline, derived from the areca catechu plant and historically consumed by various cultures, has diverse physiological effects [4,5]. It is notorious for inducing addictive behaviors and being linked to various cancers [6,7,8] primarily due to its ability to increase ROS production—a crucial factor in carcinogenesis [6]. Therefore, exploring the potential role of antioxidants in preventing and treating cancer is of significant importance. However, recent studies challenge the notion that reducing ROS generated by carcinogens always helps prevent cancer. Antioxidants, such as NAC and vitamin E, traditionally considered cancer-fighting agents, do not consistently demonstrate a reduction in cancer risk [9]. Intriguingly, some studies indicate that these antioxidants might even promote the development of certain cancers [10,11,12], leading to a paradoxical situation where they may inadvertently offer a survival advantage to cancer cells. This advantage enables cancer cells to flourish and develop resistance to treatments [13,14]. This paradox highlights the need for further research on the effects of antioxidants on cancer cells.

Prominent bioactive compounds, arecoline from the areca nut and epigallocatechin-3-gallate (EGCG) from green tea, have distinct impacts. Arecoline has been linked to the development of various cancers primarily due to its ability to increase ROS production—a crucial factor in carcinogenesis [6], while EGCG, a potent antioxidant, demonstrates selective toxicity toward cancer cells by interacting with proteins specifically overexpressed in cancer cells [15]. EGCG from green tea is a multifunctional compound showing a blend of antioxidant, anti-inflammatory, and protein-modulating activities. These diverse biochemical interactions contribute to its overall health benefits and position EGCG as a potential lead in drug design [16]. Despite their widespread use, our understanding of their combined cellular effects on cancer cells is still developing. Arecoline, derived from the areca catechu plant, has diverse physiological effects and is typically consumed by chewing betel quids, a practice prevalent in South and Southeast Asian countries such as India, Bangladesh, Myanmar, and Thailand [4,5]. Understanding the interaction between arecoline (which promotes cancer via ROS production) and antioxidants like EGCG (targeting cancer cells) is critical, especially in individuals addicted to arecoline, as it could notably affect cancer development in these populations.

The hypothesis that ROS can induce tumor cell death and reduction of ROS offers a survival advantage to cancer cells has led to studies indicating that pure antioxidants like NAC may promote certain cancers [9,10,11,12,14]. This study aims to test whether EGCG is an alternative effective antioxidant that does not protect cancer cells like NAC and whether it could synergistically enhance the cytotoxic effects of the carcinogen arecoline on cancer cells.

## 2. Methods

### 2.1. Cell Culture

PC-3 cells (ATCC CRL-1435), an androgen-independent human PC cell line [17], were used as the cell model for studying the synergistic effects of arecoline and EGCG. The cells were cultured in 100 mm dishes (GeneDireX, Inc., Taoyuan, Taiwan) using RPMI 1640 medium (Gibco-Invitrogen, Grand Island, NY, USA) containing 10% FBS (fetal bovine serum) (Thermo Fisher Scientific, Waltham, MA, USA), 100 µg/mL penicillin, and 100 µg/mL streptomycin (Sigma, St. Louis, MO, USA), and were incubated at 37 °C in a humidified atmosphere of 5% CO_2_ in a Steri-Cycle CO_2_ incubator, Model 370 (Thermo Fisher Scientific, Waltham, MA, USA).

### 2.2. Cell Treatment and Viability Assay

PC-3 cells were seeded at a density of 5000 cells/well in a 96-well plate with RPMI 1640 medium (Gibco-Invitrogen, Grand Island, NY, USA) containing 1% FBS and penicillin/streptomycin (100 µg/mL) for 24 h. Then, the cells were treated with either EGCG (10, 20, 40, and 80 µM) supplied by Sigma (St. Louis, MO, USA, product number E4143, purity ≥95%), or arecoline (100, 200, 400, 800, and 1000 µM) supplied by Sigma (St. Louis, MO, USA, product number A6134, purity >99.9% by HPLC), or with a combination of EGCG and arecoline at a fixed ratio of 1:10. For instance, if EGCG was used at a concentration of 40 µM, arecoline was correspondingly used at a concentration of 400 µM. This ratio was crucial for the analysis of synergistic effects, in line with the requirements of the CompuSyn program (https://www.combosyn.com/index.html, accessed on 17 July 2023) (Combosyn, Inc., Paramus, NJ, USA). After incubation for 48 h, the tetrazolium dye MTT (298-93-1, ≥97.5% purity, Sigma, St. Louis, MO, USA) was added to a final concentration of 0.5 µg/mL and incubated in the dark at 37 °C for 3 h. Then, the supernatant was removed and an aliquot of 100 µL of 100% DMSO (154938, ≥99.9% purity, Sigma, St. Louis, MO, USA) was added to stop the reaction and allow the insoluble formazan to dissolve in the DMSO. The absorbance was read at 570 nm using the Multiskan FC microplate photometer (Thermo Fisher Scientific, Waltham, MA, USA).

### 2.3. Determination of Synergistic Effect and Choice of Treatment Concentrations

Since cell viability is measured as the percentage of cells alive, it needs to be converted to fraction of cells affected (Fa) by subtracting from 1 to calculate combination index (CI) for assessing the synergy. First, Fa of PC-3 cells treated with either EGCG, arecoline, or a combination of fixed EGCG to arecoline concentration ratios were calculated using the format: Fc = 1 − viability. The dose–response data for single chemicals (EGCG or arecoline) and combination (EGCG + arecoline) were imputed into the CompuSyn program (https://www.combosyn.com/index.html accessed on 9 February 2024) (Combosyn, Inc., Paramus, NJ, USA), which is designed for determining the synergism, antagonism, or additive effects in treatment combinations based on the theorem of Chou-Talalay [18,19]. By running the program, the combination Index (CI) was automatically calculated, with CI values of less than 1, equal to 1, and greater than 1 indicating synergism, additive effects, and antagonism, respectively. Finally, a predicted dose–response curve (µM–Fa plot) and a predicted CI–Fa diagram were generated.

### 2.4. Reactive Oxygen Species Analysis

For measurement of intracellular ROS, 1.2 × 10^5^ cells per well were seeded in 6-well culture plates and cultured in 1% FBS medium. ROS were detected using 2-,7-dichlorofluorescin diacetate (DCFDA) as a probe, which can diffuse into cells, then deacetylated by esterase, and finally oxidized by ROS into a fluorescent compound, 2′, 7′-dichlorofluorescein. PC-3 cells were treated with arecoline alone or in combination with EGCG or NAC. After treatment, cells were washed with PBS and then incubated with 30 µM DCFDA in PBS for 30 min. After removing DCFDA, cells were trypsinized, and the ROS-generated fluorescence intensity was measured at an excitation wavelength of 485 nm and an emission wavelength of 535 nm using the Multiskan FC microplate photometer (Thermo Fisher Scientific, Waltham, MA, USA). Cells were counted using the 0.4% trypan blue exclusion method. The measured fluorescence intensities were normalized to the number of cells.

### 2.5. Flow Cytometric Analysis of Cell Cycle and Apoptosis Phases

DNA content in individual cells of different cell cycle (G1, S, G2/M) and apoptosis (sub G1) phases were analyzed using flow cytometry according to a previous publication [20]. Briefly, 6 × 10^5^ PC-3 cells were seeded in a 10-cm dish with RPMI 1640 medium for 24 h, and then treated with 400 µM arecoline, 40 µM EGCG, or a combination of both arecoline and EGCG for another 48 h. The cells were suspended by trypsinization, pelleted by centrifugation, washed with cold phosphate-buffered saline (pH 7.4), fixed in cold 70% ethanol, permeabilized with Triton X-100, treated with RNase A, and stained with propidium iodide. The DNA histograms of cell cycle distributions were determined based on DNA content by flow cytometric analysis of 10^4^ cells using CELLQuest Software on a FACSCalibur flow cytometer (BD Biosciences, San Jose, CA, USA).

### 2.6. Annexin V-Binding Assay

To more accurately quantify apoptosis levels, we employed an imaging assay using annexin V-FITC and propidium iodide (PI), utilizing the ApoDetect Annexin V-FITC Kit (Thermo Fisher Scientific, Waltham, MA, USA, catalog number 33-1200), in accordance with the manufacturer’s instructions. In summary, each sample, containing 10^5^ cells, was first washed with a binding buffer (10 mM Hepes/NaOH, pH 7.4, 140 mM NaCl, 2.5 mM CaCl_2_). Subsequently, the cells were stained with Annexin V-FITC, diluted 1/20 in the binding buffer for 10 min, followed by staining with PI (1 μg/mL) for an additional 10 min. Observations were made under a Nikon Eclipse Ti2-U Inverted Research Microscope (Nikon Corporation, Tokyo, Japan) and images were captured. The fluorescence intensities of FITC and PI signals were quantitatively analyzed using ImageJ software (Version 1.54g) (https://imagej.nih.gov/ij/ accessed on 9 February 2024).

### 2.7. Western Blot Analysis

Western blot analysis was conducted to examine the levels of cyclins, CDK, CDKI, PARP, and caspase-3 proteins. Briefly, 50 µg of protein samples were loaded onto a 12% SDS-PAGE gel and separated by electrophoresis. The separated proteins were then transferred to a PVDF membrane. The membrane was blocked for 1 h at room temperature with 5% skimmed milk in PBST and then incubated with primary antibodies at 4 °C overnight. After washing, the membrane was incubated with horseradish peroxidase-conjugated secondary antibodies and subjected to chemiluminescence detection. Protein bands were quantified using Image J software. The protein expression levels were normalized to that of actin and expressed as percentages relative to the control group.

The primary antibodies used included PARP (Cell Signaling Technology, Danvers, MA, USA, #9542), CDK1 (Cell Signaling Technology), CDK2 (Santa Cruz Biotechnology, Dallas, TX, USA, sc-163), CDK4 (Cell Signaling Technology, #12790), CDK6 (Cell Signaling Technology, #3136), cyclin D1 (Cell Signaling Technology, #2978), cyclin D3 (Cell Signaling Technology, #2936), cyclin B1 (Cell Signaling Technology, #4138), p18 (Cell Signaling Technology, #2896), p21 (Cell Signaling Technology, #2947), p27 (Cell Signaling Technology, #3686), and β-actin (Cell Signaling Technology, #8457). The secondary antibodies were horseradish peroxidase-conjugated Donkey anti-goat IgG-HRP (Santa Cruz Biotechnology, sc-2020), Donkey anti-rabbit IgG-HRP (Santa Cruz Biotechnology, sc-2313), and Goat anti-mouse IgG-HRP (Santa Cruz Biotechnology, sc-2005).

### 2.8. Statistical Analysis

Statistical comparisons between indicated groups in figures were performed using the Student’s *t*-test. Significance was marked as *p* < 0.05 (*) for significant and *p* < 0.01 (**) for highly significant differences. Data are expressed as mean ± standard deviation (SD), detailing the comparisons across the indicated groups.

## 3. Results

### 3.1. EGCG Synergistically Enhanced Cytotoxic Effects of Arecoline

**Figure 1:**
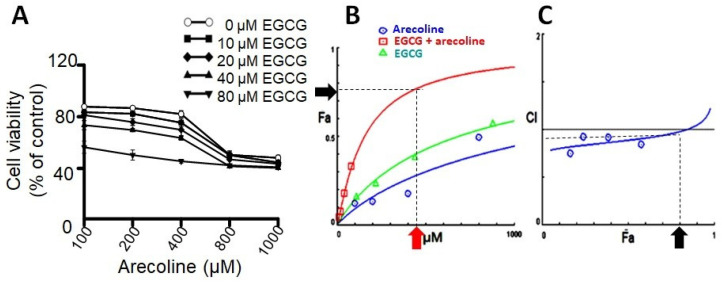
The results of combinational effects of EGCG and arecoline on cell viabilities and evaluation of their synergism. (**A**) Concentration–viability plots for individual chemicals (treated with EGCG or arecoline) and their combinations. The concentrations of EGCG used were 10, 20, 40, and 80 µM and those of arecoline were 100, 200, 400, 800, and 1000 µM. Cell viability was measured using the MTT assay. (**B**) Dose–effect curves showing percent fraction of cells (Fa) affected by increasing and continuing concentrations of EGCG, arecoline, or their combination, are displayed in green, blue, and red, respectively. The near-plateau dose (440 µM) and corresponding effect (75% Fa) are indicated by the red arrow and the black arrow, respectively. (**C**) Combination index curve calculated using the CompuSyn Software, showing synergistic effects between EGCG and arecoline in a wide range of Fa (0%~80%). The black arrow indicates that 75% Fa is the maximal synergistic effect with CI < 1.

Prior research has indicated that testosterone may facilitate the growth of prostate cells, hence impeding the cytotoxic efficacy of arecoline [21]. In order to exclude the confounding variable, PC-3 cells were employed as the experimental model in this investigation due to their lack of androgen receptor expression and hormone insensitivity [17]. In order to examine the synergistic effects of arecoline and EGCG, PC-3 cells were exposed to different concentrations of a combination of EGCG and arecoline. As depicted in Figure 1, the viability of cells was diminished in a concentration-dependent way by the presence of arecoline. Furthermore, the survival curve exhibited a pronounced downward and leftward shift when arecoline was administered in combination with EGCG (Figure 1A), suggesting a synergistic effect. The study of combined effects of EGCG and arecoline was further performed using the CompuSyn software (Combosyn, Inc. NJ, USA.), which was developed based on the Chou-Talalay theory and can accurately predict the dose–effect relationship and precisely analyze the synergistic (CI < 1), additive (CI = 1), or antagonistic (CI > 1) effects of drug combinations [18,19]. As shown in the Dose–Fa plot depicted in Figure 1B, it is evident that the combined-treatment curve (represented by the red line) exhibited a significant increase in the fraction of affected cells (Fa) compared to the individual curves of arecoline (blue line) and EGCG (green line). Moreover, it reveals that synergistic effects (indicated by CI values below 1) can be exhibited over a wide range of Fa values, spanning from 0% to 75% (Figure 1C).

Upon examining the Dose–Fa and CI–Fa plots, we noticed that Fa level of 0.75 is near the maximal synergistic cytotoxic effect associated with a CI index below 1 (blue arrow in Figure 1C), which was generated by a combination of 400 µM arecoline and 40 µM EGCG (red arrow in Figure 1A). We therefore chose these concentrations (440 µM total) for the subsequent experiments to explore the underlying cellular and biochemical mechanisms of synergism.

### 3.2. EGCG and NAC Inhibited Arecoline-Generated ROS but Had Different Effects on PC Cell Viability

Given the potent antioxidant properties of EGCG, which effectively counteract ROS to mitigate oxidative stress [22], it is interesting to compare the impacts of EGCG (dissolved in DMSO) and NAC (dissolved in phosphate-buffered saline) on ROS generation and cellular viability in PC cells exposed to arecoline, with DMSO serving as the control for EGCG-treated cells and PBS as the control for NAC-treated cells. As illustrated in Supplementary Figure S1, arecoline induced a substantial increase in ROS production within PC cells. However, this rise in ROS levels can be counteracted by concurrent treatment of either NAC or EGCG (Supplementary Figure S1A), underscoring the efficacy of both compounds as antioxidants. Notably, while the antioxidant NAC attenuated the cytotoxicity triggered by arecoline, EGCG, with its antioxidative effect, acted synergistically with arecoline to amplify the cytotoxic effect on PC cells (Supplementary Figure S1B). It is important to note the distinct concentrations used in our experiments: 1.5 mM for NAC and 40 µM for EGCG. The selection of these concentrations is consistent with those reported in the existing literature. Specifically, a higher concentration of NAC is required (in the millimolar range), while for EGCG, only micromolar levels are necessary [23,24]. This disparity reflects the inherent biological properties of these compounds. This observation suggests that EGCG operates through mechanisms beyond its antioxidant capabilities, thereby countering its antioxidant-mediated support for cancer cell survival and even enhancing arecoline-induced decline in the viability of PC cells.

### 3.3. EGCG Synergistically Increased Apoptotic Events in Arecoline-Treated PC Cells

**Figure 2:**
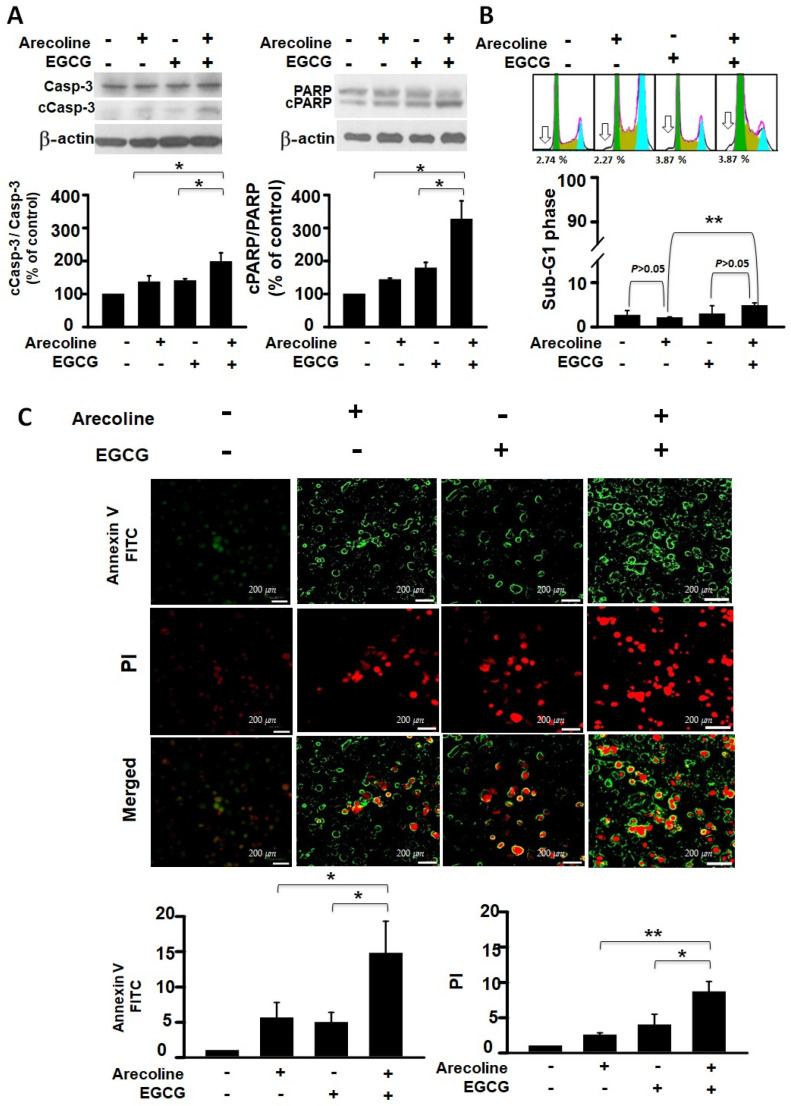
Synergistic Impact of Epigallocatechin Gallate and Arecoline on Apoptotic Markers: Caspase 3 Activation, PARP Cleavage, Chromatin Fragmentation, and Phosphatidylserine Externalization. This study examines apoptosis in four groups: untreated, 40 µM EGCG, 400 µM arecoline, and their 440 µM combination, aiming to uncover EGCG and arecoline’s synergistic effects on apoptotic markers. (**A**) Western blot analysis shows the expression of procaspase 3 (Casp-3) and cleaved caspase 3 (cCasp-3) on the left side of the figure, as well as the expression of full-length PARP (PARP) and cleaved PARP (cPARP) proteins on the right side of the figure. The upper panels of both sides show representative immunoblots of the levels of full-length and cleaved proteins, while the lower panels quantitatively present the statistical assessment of cleavage levels of the proteins (full-length protein/cleaved protein ratios). (**B**) Flow cytometry data reveals the cumulative fluorescence signal intensities and the corresponding cell count percentages within the sub-G1 fraction of the cell cycle. The upper panel shows a representative set of histograms obtained from flow cytometry experiments, with hollow arrows indicating the sub-G1 phase regions. The lower panel quantifies the statistical analysis of the proportion of cells residing in the sub-G1 phase. (**C**) Annexin V and Propidium Iodide Staining Validates Phosphatidylserine Externalization and Cell Viability. The left panels display fluorescence microscopy images showing cells stained with Annexin V-FITC and Propidium Iodide (PI), indicating externalized phosphatidylserine and membrane integrity, respectively. The right panels provide quantitative analysis of Annexin V-FITC and PI staining. Notably, it is observed that there is a significant difference (*p* < 0.05 or *p* < 0.01) between the indicated experimental groups. This data collectively highlights the synergistic effects of EGCG and arecoline on phosphatidylserine externalization, cell membrane integrity loss, and the fragmentation of Caspase-3, PARP proteins, and nuclear DNA. * and ** indicate *p* < 0.05 and *p* < 0.01 for comparisons among the indicated groups, respectively.

To determine whether arecoline reduces PC cell viability through apoptosis and whether EGCG further decreases viability by enhancing that mechanism, we analyzed this from both protein and nucleic acid perspectives. The events of cell apoptosis, including procaspase 3 (Casp-3) activation, PARP cleavage, chromatin fragmentation, and phosphatidylserine (PS), flip were assessed using western blot analysis, flow cytometry, and fluorescence microscopic imaging. We examined the differential effects of arecoline alone, EGCG alone, and their combined action on these apoptosis events. Our results indicate that both arecoline and EGCG individually promoted cleavage of Casp-3 and PARP (in the left and right panels of Figure 2A, respectively). The combination of arecoline and EGCG synergistically increased the degree of cleavage (Figure 2A). A similar phenomenon was observed in DNA fragmentation, where flow cytometry analysis of sub-G1 phase cells revealed that both arecoline and EGCG alone increased the proportion of cells with fragmented DNA. Notably, the combination of arecoline and EGCG further elevated the proportion of sub-G1 phase cells (Figure 2B). Furthermore, fluorescence microscopic imaging provided additional insight into the early and late stages of apoptosis. Annexin V-FITC staining, displayed in green, indicated the externalization of phosphatidylserine, a hallmark of early apoptosis (Figure 2C, left panels). Conversely, PI staining, evident in red, denoted a loss of cell membrane integrity, characteristic of late apoptosis or necrosis (Figure 2C, right panels). The merged images underscored the presence of cells at various apoptotic stages: early apoptotic cells stained green, late apoptotic cells stained both green and red, and necrotic cells stained predominantly red. These observations underscore the synergistic augmentation of apoptotic signals when arecoline and EGCG are combined, as evidenced by enhanced phosphatidylserine externalization and cell membrane permeabilization. This multifaceted approach confirms that the additional decrease in cell viability caused by EGCG is likely through an amplified apoptosis mechanism.

### 3.4. EGCG Restores Cell Cycle Progression by Redirecting Cells from Arecoline-Induced Cycle Arrest towards Apoptosis

**Figure 3:**
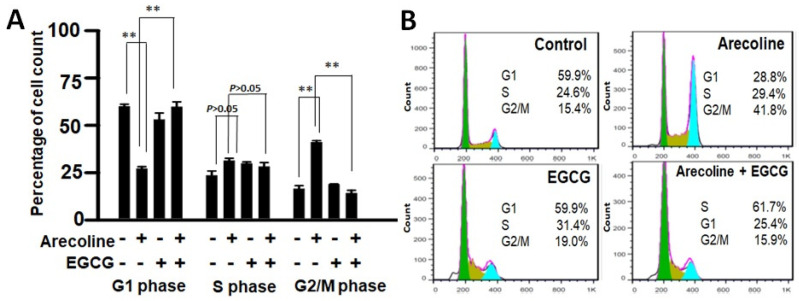
Flow cytometry data showing distribution of cell populations in the cell cycle of PC cells treated with arecoline (400 µM), EGCG (40 µM), and/or their combination (440 µM total). (**A**) Bar chart showing the percentage of cells counted in G0/G1, S, and G2/M phases. Data are expressed as the mean ± SEM. ** indicate *p* < 0.01 for comparisons among the indicated groups, respectively. (**B**) A representative flow cytogram showing the distribution of cells in the cell cycle. Areas of green, olive, and blue represent percentages of cells in G0/G1, S, and G2/M phases, respectively. Note that G2-arrest was induced in the arecoline-treated group, showing a significant increase in G2 count together with a decrease in G1 count compared to the non-treated group. Of note, co-treatment with EGCG abolished the arrest and resumed cell cycle progression, displaying reversing of the cell counts to a non-arrest state.

Cell cycle arrest involves a temporary halt in biochemical processes, signaling cells to cease dividing and undergo repair or death. To examine the combined effects of EGCG and arecoline on the cell cycle, we utilized flow cytometry analysis to assess DNA content in cells at distinct cell cycle phases (G1, S, G2/M). As depicted in Figure 3, arecoline significantly induced cell cycle arrest, halting cells at the G2/M phase (Figure 3A). This resulted in an increased cell count at G2/M, accompanied by a reduced count in the subsequent G1 phase. Intriguingly, the co-treatment of EGCG appeared to counteract this arrest, allowing the resumption of cell cycle progression. This is evident from the reversion of cell counts to a state resembling non-arrest conditions (Figure 3A). Following a cell cycle arrest, cells can be directed towards repair or apoptosis, depending on their repair potential. Our data, which clearly demonstrated that EGCG synergistically reduced viability (Figure 1 and Supplementary Figure S1) and augmented apoptosis (Figure 2) in the PC cells, suggest that EGCG enhanced the apoptotic process, thereby reducing G2/M proportion by converting the excess viable G2 cells into dead apoptotic cells.

### 3.5. EGCG and Arecoline Synergistically Impacted the Abundance of Cyclins, Cyclin-Dependent Kinase (CDK), and CDK Inhibitors

Cell cycle stages are regulated through a cooperative interaction between cyclins and cyclin-dependent kinases (CDKs) [25]. The levels of cyclin proteins undergo periodic production and breakdown during the cell cycle, playing a crucial role in controlling the activities of CDK during the course of the cycle. Beyond the CDK–cyclin interaction, CDK inhibitors (CDKIs) serve as a braking system [25], critically halting the activity of CDK–cyclin complexes to prevent the continuation of erroneous cell cycle events. To unravel the biochemical mechanisms, we examined expressional levels of critical cyclins, CDKs, and CDKIs that could contribute to the synergistic interaction between EGCG and arecoline.

**Figure 4:**
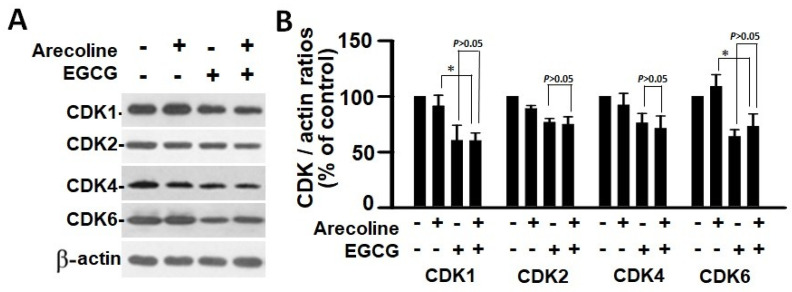
Cyclin-dependent kinase expression in PC cells treated with arecoline (400 µM), EGCG (40 µM), and their combination. (**A**) Representative Western blot images illustrating the expression profiles of CDK1, CDK2, CDK4, and CDK6 in PC cells treated with arecoline, EGCG, and a combination of arecoline and EGCG. Expression levels were normalized to that of β-actin as the loading control, with untreated cells serving as the baseline for comparison. (**B**) Quantitative bar graph depicting the statistical data for the expression levels of CDK1, CDK2, CDK4, and CDK6. The presented values are mean ± SEM. Significance is indicated by * *p* < 0.05 among the indicated groups.

**Figure 5:**
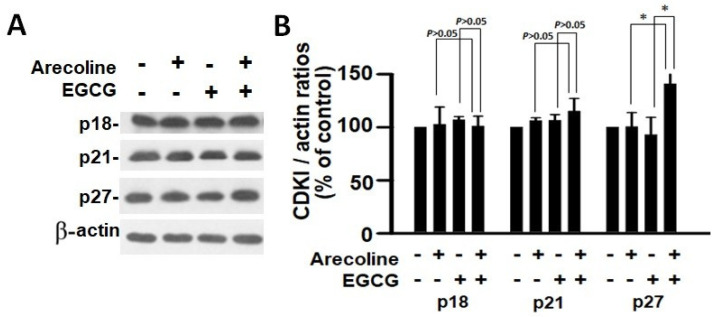
Analysis of the expression of cyclin-dependent kinase inhibitors in PC cells exposed to arecoline (400 µM), EGCG (40 µM), and their combination. (**A**) Representative Western blot images illustrating the expression levels of p18, p21, and p27 in PC cells subjected to treatment with arecoline, EGCG, and combined treatment. β-actin was utilized as the loading control, and an untreated group was used as the treatment control. (**B**) Quantitative bar chart depicting statistical data for p18, p21, and p27 expression levels. The presented values represent the mean ± SEM, normalized to control conditions. Significance is indicated by * *p* < 0.05 among the indicated groups.

**Figure 6:**
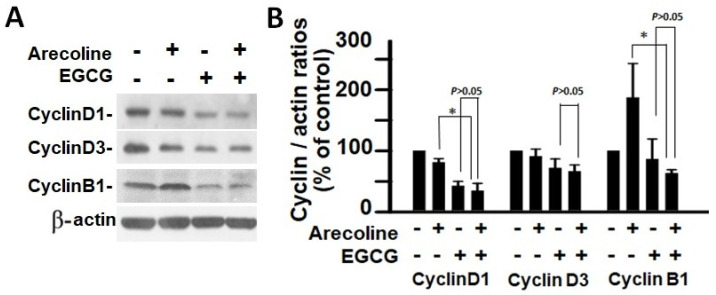
Analysis of cyclin expression in PC cells after treatment with arecoline (400 µM), EGCG (40 µM), and their combination. (**A**) Western blot results showcasing the expression patterns of cyclin D1, cyclin D3, and cyclin B1 in PC cells exposed to arecoline, EGCG, or a combination of arecoline and EGCG. *β*-actin was used as the internal control for expression normalization, and untreated cells were utilized as treatment references. (**B**) Statistical representation in the form of a bar chart, illustrating the quantified levels of cyclin D1, cyclin D3, and cyclin B1. Data are expressed as the mean ± SEM and have been normalized to control conditions. * indicates *p* < 0.05 for comparisons among the indicated groups, respectively.

According to the findings, treatment with arecoline had no discernible effect on the expression levels of CDKs (Figure 4) and CDKIs (Figure 5). However, it significantly increased the amount of cyclin B1, which plays an essential function in cell cycle regulation (Figure 6). Since the breakdown of cyclin B1 is a prerequisite for the transition from the G2 phase to the M phase, the significant buildup of cyclin B1 could explain why arecoline inhibited the progression of the cell cycle. This is demonstrated by the observed arrest of the cell cycle at the G2/M phase in the arecoline-treated cells.

**Figure 7:**
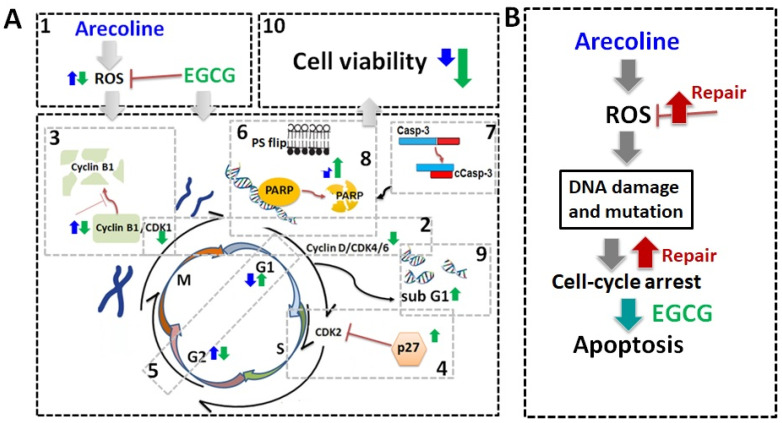
Schematic summary of biochemical mechanisms and synergistic effects of EGCG on arecoline-treated cancer cells. (**A**) Schematic diagrams of biochemical mechanisms. EGCG not only lowers ROS generation [1] but also causes pan-suppression of cyclin–CDK [2], degradation of cyclin B1 [3], and increase in p27 [4], thus leading to cell cycle arrest and guiding cells from G2 arrest [5] towards apoptosis. This intensifies the externalization of PS [6] and fragmentation of Casp-3 [7], PARP [8], and DNA [9], culminating in increased cell death [10]. The effects of arecoline and EGCG are depicted with blue and green arrows, respectively. (**B**) Arecoline-induced ROS generation can be counteracted by promoting cancer cell survival but can also cause molecular damage, leading to cell cycle arrest. Some cancer cells may survive with exacerbate mutations via repair mechanisms. Synergistic effects of EGCG and arecoline enhance apoptosis in the damaged cancer cells, thus reducing cancer progression.

In contrast, EGCG caused a substantial reduction in various cyclins and CDKs (Figure 4 and Figure 5), leading to an impact on all stages of cell cycle without specific arrest at any stage. In addition, although EGCG treatment alone did not alter CDKIs (p18, p21, and p27), it notably elevated p27 levels in combination with arecoline treatment (Figure 3). Given that p27 functions as a CDKI impeding CDK2/cyclin E activity at the G1 checkpoint that detects DNA damage (as illustrated in Figure 7A), the enhancement of p27 levels by EGCG might augment the capability to sense DNA damage induced by arecoline. Taken together, the pan-suppression of cyclins and CDKs and the specific elevation of p27 levels by EGCG underlie the profound synergistic effects of EGCG on reducing cell viability and increasing apoptosis (Figure 1 and Figure 2, and Supplementary Figure S1).

## 4. Discussion

To date, arecoline has been confirmed as a carcinogen, inducing gene mutations and molecular damage through the induction of oxidative stress, leading to cell cycle arrest [26,27]. Our data provide conclusive evidence that EGCG synergistically inhibited both cell viability and ROS production in arecoline-treated cells, as summarized in Figure 7. The synergy resulted from the accumulation of cyclin B1 and p27, leading to cell cycle arrest. This directed cells from G2 arrest towards apoptosis and intensified the fragmentation of Casp-3, PARP, and DNA, ultimately resulting in increased cell death. A comparison with NAC highlights the advantage of EGCG as an antioxidant adjuvant for cancer prevention and treatment, as it not only lowers ROS levels but also synergistically enhances arecoline-induced cytotoxicity.

Many carcinogens and chemotherapeutic chemicals cause cell damage, evoking cell arrest that leads to apoptosis [28,29,30]. However, cancer cells develop mechanisms to repair the damages and pass the cell cycle checkpoints to continue proliferation [31]. Cell cycle arrest results in either programmed cell death or the activation of DNA repair mechanisms, depending on the ability of self-repair [32,33]. Our data indicate that, as suggested in Figure 7B, in the absence of EGCG, the PC-3 cell cycle was halted and could not progress to apoptosis in the presence of arecoline only. It can be predicted that the cell cycle arrest caused by arecoline increases the chances of ROS-induced mutagenesis, potentially giving rise to more aggressive cancer traits. When cancer cells were treated with both arecoline and EGCG, the cells were more likely to undergo apoptosis rather than just enter a state of cell cycle arrest (Figure 7B). This combined treatment reduced opportunities for cellular repair and decreased the survival of cancer cells, thereby reducing the risk of accruing additional DNA mutagenesis. This interplay underscores the potential of employing EGCG to mitigate the carcinogenic impact of arecoline, offering a promising advantage in cancer treatment.

Arecoline has been identified as a major factor causing numerous disorders, including neurotoxicity and cancer [6,7,8,34,35,36,37,38]. Regarding cancer, arecoline-generated ROS can affect the expression of various cell cycle regulators (CDKs, cyclins, and CDKI), leading to unrestrained DNA replication and uncontrolled cell cycle transition, promoting cancer viability and growth [39]. While antioxidants are typically viewed as protective agents against ROS-induced DNA damage and cancer progression [40], ROS are generated by various cellular processes and can cause damage to lipids, proteins, and DNA. Excessive ROS can initiate cancer development through DNA damage by oxidizing DNA bases, inducing gene mutations, activating oncogenes, and inhibiting tumor suppressor genes [1,2]. In terms of cancer cells, this foundational damage promotes a more malignant condition [6] in which ROS continuously damage cellular components, resulting in a more severe state [41,42,43]. Consequently, ROS transform benign cancer into malignant cancer by excessively activating epithelial–mesenchymal transition signaling pathways [3]. These pathways result in the loss of cell–cell junctions, the remodeling of the cytoskeleton, and the degradation of the extracellular matrix, all of which allow cancer cells to migrate and invade. In this context, it is believed that antioxidants act as a molecular barrier against cancer by neutralizing ROS and mitigating these harmful effects, providing significant health benefits.

Our data demonstrated that pure antioxidants, such as NAC, increased cell viability by protecting cancer cells from ROS-induced damage, consistent with previous findings that NAC and vitamin E do not reduce the risk of cancers [9] and may even support the development of melanoma, lung cancer, and intestinal tumors [10,11,12]. In contrast, antioxidants with other biological effects, like melatonin and EGCG, can counteract arecoline-induced oxidative damage and other premalignant conditions [9,13]. Our study showed that EGCG not only reduced ROS generation but also promoted apoptosis of cancer cells. As a green tea polyphenol with potent antioxidant and ROS-counteracting abilities, EGCG has shown promise in cancer therapy. Its effect arises not just from its natural antioxidant properties but also its differential effects on normal and cancer cells. While EGCG efficiently neutralizes ROS to reduce oxidative stress—a known factor in cancer development—it also targets other proteins within cells to inhibit overgrowth, halt the cell cycle, and induce apoptosis [15]. Importantly, these interactions are primarily detrimental to cancer cells as EGCG is more likely to impede the activity of signal transduction factors frequently over-activated in cancer. Interestingly, chemotherapeutic chemicals also kill cancer cells by elevating ROS levels [44,45].

A limitation of our study is the exclusive use of the PC-3 cell line, which may not represent the full spectrum of cancer cells. Recognizing the crucial value of primary cultures, which more closely mimic the complex biological and molecular environment of tumors in vivo, future research should aim to include these to better mirror real patient conditions. In summary, recent research suggests that reduction of ROS could protect cancer cells from ROS-induced damage. EGCG not only efficiently neutralizes ROS generation but also reduces the survival of cancer cells. Consequently, EGCG could potentially serve as a more effective preventive or therapeutic adjunct for cancer based on its diverse mechanisms.

## Data Availability

All the data and materials in the current study are available upon reasonable request.

## Supplementary Materials

The following supporting information can be downloaded at: https://www.mdpi.com/article/10.3390/cimb46020098/s1.
