# Supplementary material for: Epigallocatechin-3-gallate Synergistically Enhanced Arecoline-Induced Cytotoxicity by Redirecting Cycle Arrest to Apoptosis
Source: Curr Issues Mol Biol. 2024 Feb 14;46(2):1516–29. doi: 10.3390/cimb46020098 (PMC10887523; doi:10.3390/cimb46020098)
Supplement: Supplementary file 1 [file cimb-46-00098-s001.zip › cimb-2778362-supplementary.pdf]

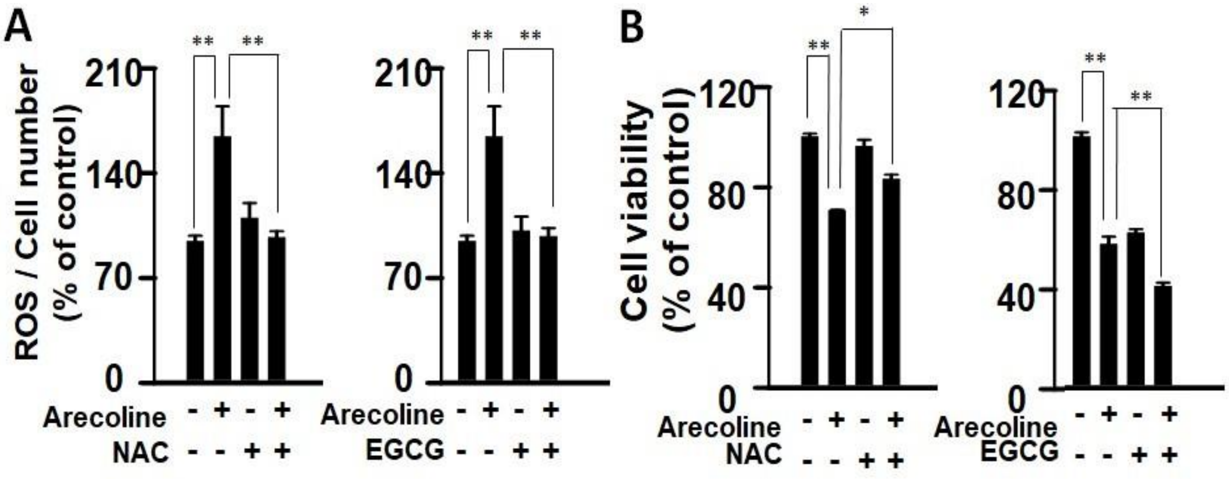

Supplementary Figure S1. Differential Effects of EGCG and NAC on Arecoline-Triggered ROS Generation and Reduced Cellular Viability. (A) The effects of 1.5 mM NAC and 40 μM EGCG on arecoline-induced ROS levels in PC cells were examined. ROS production was measured using the 2-,7-dichlorofluorescein diacetate method. Notably, both NAC and EGCG exhibited similar reductive effects on ROS levels when treated with arecoline, as compared to the cells treated with arecoline alone. (B) The effects of 1.5 mM NAC and 40 μM EGCG on the diminished viability of PC cells due to arecoline exposure were assessed using the MTT assay. Remarkably, NAC counteracted the cytotoxic effects of arecoline, while EGCG synergistically intensified the cytotoxic impact of arecoline on PC cells. * and ** indicate $P < 0.05$ and $P < 0.01$ for comparisons among the indicated groups, respectively.
